# Exploring the relationship between core stability and vertical jump in recreationally active male college students based on a suite of novel core stability assessments

**DOI:** 10.1016/j.heliyon.2024.e25236

**Published:** 2024-01-28

**Authors:** Jay Lee, Liang Wang, Xiuli Zhang

**Affiliations:** a Faculty of Education, University of Macau, Macao, China; b School of Physical Education & Sports Science, South China Normal University, Guangzhou, China

**Keywords:** Core stability, Vertical jump, Correlation analysis, Sports improvement, Movement quality

## Abstract

Various assessments have contributed to inconsistent findings regarding the correlation between core stability and vertical jumps. Therefore, this study aimed to re-examine this correlation based on novel core stability assessments. Twenty-one recreationally active male college students (age, 21.7 ± 2.1 years; stature, 174.9 ± 6.7 cm; body mass, 67.7 ± 7.8 kg; leg length, 88.9 ± 4.8 cm; arm length, 87.8 ± 4.0 cm) participated in this experiment. Core stability was divided into static and dynamic core stabilities, with the static core stability measured using the Eight-Level Prone Bridge and Five-Level Side Bridge tests and the dynamic core stability measured using the Y Balance Test (YBT). These tests comprehensively evaluate core stability as it is defined. Kinematic and kinetic data on vertical jumps were collected to provide process information beyond the outcome performance. Subsequently, these data were correlated with core stability for a deeper insight into the relationship between core stability and the process and outcome performance of vertical jumps. The main results revealed that the Eight-Level Prone Bridge demonstrated moderate to substantial correlations with Δ $\bar{F_{y}}$, Δ $I_{y}$, $\Delta D_{left\;knee\;z}$, and $\Delta D_{left\;ankle\;y}$ (−0.62 ≤ r ≤ 0.52); the Five-Level Side Bridge exhibited moderate correlations with Δ $\bar{F_{x}}$, Δ $\bar{F_{y}}$, Δ $I_{x}$, Δ $I_{y}$, $\Delta D_{left\;knee\;z}$, and $\Delta D_{left\;ankle\;y}$ (−0.52 ≤ r ≤ 0.59); YBT displayed moderate correlations with $\bar{F_{z}}$, $\bar{F_{left\;z}}$, Δ $D_{left\;ankle\;y}$, Δ $D_{right\;ankle\;y}$, Δ $D_{left\;ankle\;z}$, Δ $D_{right\;ankle\;z}$, NΔ $\bar{T_{ankle\;y}}$, and N $\bar{T_{left\;ankle\;z\;}}$ (−0.54 ≤ r ≤ 0.54) during the propulsive phase of vertical jumps. However, no significant correlations were observed between static/dynamic core stability and jumping height. Therefore, individuals with greater core stability should experience improved process performance (better movement quality), although this benefit is ineffective in translating into jumping height improvement due to impaired explosive features. Coaches may consider core stability in training to trigger an improved process performance of the vertical jump when the technique is the key issue to be solved, although future studies are required to verify this further.

## 1 Introduction

Core stability is defined as the capacity of the joints within the core zone—encompassing the shoulder joint, trunk, hip joint, and pelvis, inclusive of an intricate network of the subsidiary neural and connective tissues such as muscles and ligaments—to withstand external perturbations (e.g. gravity) and maintain a neutral position (reasonable posture) in the frontal, sagittal, and transverse planes, whether in a stationary or dynamic state [[1], [2], [3], [4], [5], [6], [7], [8], [9], [10]]. Therefore, it can be divided into static and dynamic core stabilities [[11], [12], [13]].

Theoretically, core stability plays a critical role in sports performance improvement and injury prevention [1,4,5,14,15]. Exceptional core stability empowers the effective control of the trunk and posture stabilisation and provides a fundamental groundwork for generating and transmitting force production between the upper and lower extremities. Consequently, this optimisation of force or energy production and transmission should enhance vertical jump performance and diminish the possibility of sports-related injuries [1,5,6,11,16,17]. For instance, Kabadayi et al. [18] noted that an 8-week core training on karate practitioners could significantly improve their core endurance and countermovement jump (CMJ) ability. Guo et al. [4] demonstrated that dynamic core strength could facilitate force and power transfer to improve countermovement jump performance. Similarly, in a study on football players, Nesser et al. [16] reported a significantly moderate correlation between core stability and CMJ ability. In summary, the effect of core stability on vertical jump performance has gained popularity among athletes.

However, there is no consensus on the effect of core stability on vertical jump performance because not all studies have observed a significant improvement [10,12,19]. In a study of 83 female athletes from hockey, netball, running, soccer, and tennis, Bruin et al. [20] applied core strength and endurance to measure core stability and found that negligible or weak correlations were observed between core stability and vertical jump. Similarly, Schilling et al. [10] demonstrated that although a 6-week core training could be conducive to core strength and endurance improvement, it was ineffective in improving vertical jump performance.

Inconsistent findings can be attributed to the absence of a universally accepted ‘gold standard’ method for assessing core stability, as different studies have used different measurements [17,21]. For instance, some studies have utilised assessments developed from McGill's core muscle endurance tests [10,16,19,20,22,23], whereas others have assessed core stability from five distinct perspectives: core strength, core endurance, flexibility, motor control, and functionality [4,21,24]. Although these approaches are valid in specific contexts [6,21], they do not comprehensively represent core stability. The former methods predominantly focus on isometric muscle contraction or endurance of the trunk musculature, particularly in patients with low back pain [6,16,19,22,23]. Conversely, the latter methods tend to conflate the five components mentioned above with core stability (i.e. the five components have parallel relationships, not inclusive relationships). Instead, the Eight-Level Prone Bridge, Five-Level Side Bridge, and Y Balance Tests require participants to sustain their joints in a functional anatomical position in the core region while the supporting base is reduced. These tests satisfy the definition of core stability based on static and dynamic measurements. Therefore, in this study, they were applied as a novel combination to evaluate core stability.

Given that vertical jump is a basic but pivotal indicator of an individual's power ability, its improvement is closely related to many athletic performance enhancement and sports injury prevention [[25], [26], [27], [28], [29]]. Moreover, previous studies have only focused on core stability and athletic performance (outcome) and, neglected the process performance of vertical jumps [4,14,18,20]. An insignificant correlation between core stability and athletic performance does not mean that core stability is ineffective in improving process performance (i.e., movement quality). Therefore, this study aimed to re-evaluate core stability and vertical jump association based on novel core stability assessments, with a comprehensive analysis of vertical jumps to decipher the association.

## 2 Materials and methods

### 2.1 Participants

This study was approved by the Human Research Ethics Committee of South China Normal University (SCNU-SPT-2020-010). Recruitment notices, including the study purposes, brief study procedures, and qualifying criteria, were disseminated at the university through flyers to recruit recreationally active male college students interested in this study. The inclusion criteria were as follows: (1) regularly performing moderate-intensity exercises (or higher intensity) no less than thrice a week with at least an hour apiece, (2) free from lower back and extremity injuries within the past 6 months, and (3) age between 18 and 30 years. Participants who met any of the following criteria were excluded: (1) professional student-athletes from the school's sports team, (2) body mass index ≥24 kg/m^2^, and (3) any movement disorders. After the screening process, 21 recreationally active male college students (age, 21.7 ± 2.1 years; stature, 174.9 ± 6.7 cm; body mass, 67.7 ± 7.8 kg; leg length, 88.9 ± 4.8 cm; arm length, 87.8 ± 4.0 cm) were included. All participants were right-extremity dominant and free from lower back and extremity injuries or diseases within the past six months. Before the experiment, the participants received comprehensive instructions regarding the study procedures and expectations, and signed an informed consent form, which included permission for the utilisation of their images.

### 2.2 Procedures

**Fig. 1:**
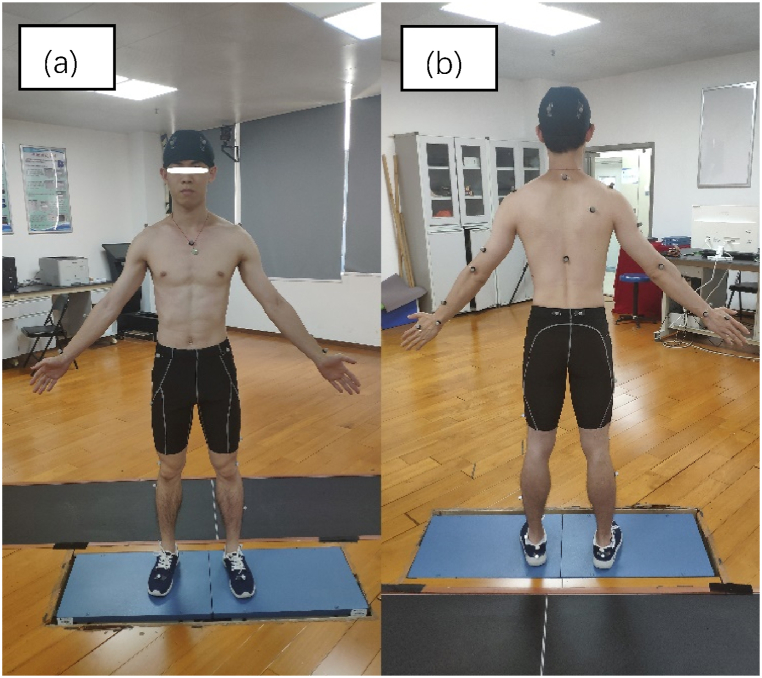
Retroreflective markers on body landmarks. (a) Front view; (b) Back view.

A uniform sports kit was used before the experiment [Fig. 1 (a)-(b)]. The following tests were sequentially performed in a biomechanics laboratory. (1) Anthropometric measurements, including stature, body weight, leg length, and arm length, were performed by the same researcher. Stature and body weight were measured using an Infrared Height and Weight Tester (BYH01, Hochoice, China). Leg length was defined as the distance between the anterior superior iliac spine and the medial condyle of the ankle joint, whereas arm length was defined as the distance between the spinous process of the seventh cervical vertebra and the middle fingertip. (2) The Eight-Level Prone Bridge Test, Five-Level Side Bridge Test, and Y Balance Test were performed after a 5-min warm-up following a ‘quick warm-up cardio workout’. This warm-up workout included 10 different exercises (boxer shuffle, overhead reach + stretch, high knee march, torso twists, toe touch kicks, full torso circles, lateral step toe touches, squats, jumping jacks, high knees) with 30-s apiece. (3) A total of 39 retroreflective markers were applied to the participants and inspected by a biomechanics assistant [Fig. 1 (a)–(b)] before performing CMJ and single CMJ (SCMJ) in sequence for sports biomechanics measurements. The whole testing process cost approximately 2 h.

### 2.3 Core stability measures

A lack of evidence regarding the effectiveness for athletic performance improvement is mainly due to a limited number of appropriate tests evaluating core stability [17]. Furthermore, a lack of appropriate tests is mainly because different studies have different interpretations of core stability [17]. For instance, some practitioners may regard core stability as the endurance or strength level of specific muscle groups within the lumbopelvic-hip complex, primarily focusing on preparing individuals for daily tasks without discomfort or maintaining proper posture under minimal external pressure [1,6,11]. However, these perspectives fall short of meeting the requirements of athletic performance, where both athletic prowess and injury prevention hold high priorities.

#### 2.3.1 Bridge tests

The Eight-Level Prone Bridge and Five-Level Side Bridge tests required the participants to maintain the functional anatomical alignment of joints within the core region while the support base was reduced. The longer they could maintain this alignment, the higher were their scores. During the bridge tests, stabilisers in the core region were maximally activated to maintain stability in the frontal, sagittal, and transverse planes. Therefore, these tests effectively assessed the ability of the core to resist flexion, extension, rotation, and lateral flexion [30]. Additionally, the Eight-Level Prone Bridge and Five-Level Side Bridge tests have been proven as reliable and easy methods for assessing core musculature function [21,[31], [32], [33], [34], [35]]. Therefore, these tests were employed to evaluate the participants’ static core stability.

All participants underwent a 10-min instructional session, followed by supervised practice led by assistants. Subsequently, the participants performed the Eight-Level Prone Bridge and the Five-Level Side Bridge tests sequentially, with a 5-min rest period allocated between each bridge test. In each bridge test, the participants were required to maintain their position for as long as possible, under the supervision of three research assistants stationed at the front, side, and above the participant. The test was terminated when the participants could no longer sustain the required position after being prompted thrice to do so by the assistants. The time was recorded in seconds using a stopwatch.

**Fig. 2:**
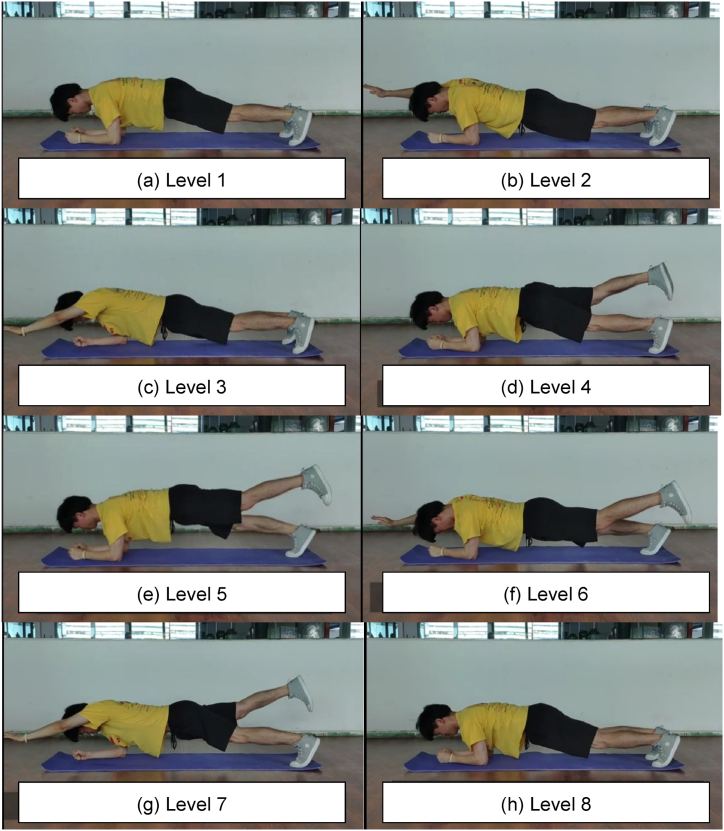
Eight-Level Prone Bridge test. (a) Level 1 starts in a prone position, with elbows and toes supporting; (b) Level 2, the right arm is raised and extended forward; (c) Level 3, put down the right arm while raising and moving forward the left arm; (d) Level 4, put down the left arm while raising and moving backward the right leg; (e) Level 5, put down the right leg while raising and moving backward the left leg; (f) Level 6, raise and move forward the right arm based on Level 5; (g) Level 7, put down the right arm and left leg while raising and extending the opposite arm and leg; (h) Level 8 is the same as Level 1.

In the Eight-Level Prone Bridge test [Fig. 2 (a)-(h)], the participants began in the prone position, supporting themselves on their elbows and toes. This level lasted for 60s [Fig. 2 (a)]. At Level 2, the participants raised their right arm off the floor and extended it forward, holding this posture for 15 s [Fig. 2 (b)]. At Level 3, the right arm returned to the starting position while the left arm was raised and extended forward for another 15 s [Fig. 2 (c)]. At Level 4, the left arm was returned to the starting position while the right leg was raised and extended backward for another 15 s [Fig. 2 (d)]. At Level 5, the right leg returned to the starting posture while the left leg was raised and extended backward for another 15 s [Fig. 2 (e)]. At Level 6, the right arm was raised forward based on Level 5 for another 15 s [Fig. 2 (f)]. At Level 7, the right arm and left leg returned to the starting posture, while the opposite arm and leg were raised and extended for another 15 s [Fig. 2 (g)]. Finally at Level 8, the arm and leg were placed back in the starting position and held for another 30 s [Fig. 2 (h)].

**Fig. 3:**
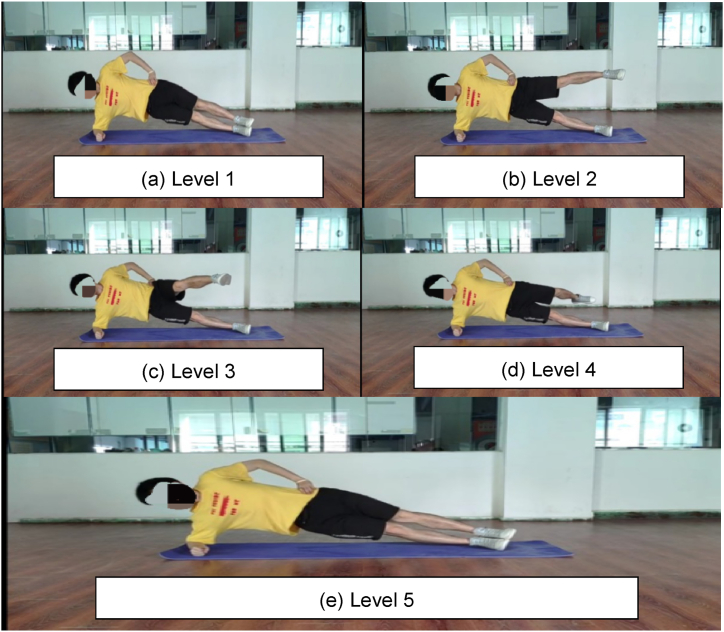
Five-Level Side Bridge test. (a) Level 1 starts in a side-lying position with one elbow and foot supporting. The supporting elbow is flexed at 90°, and the leg is extended to maintain alignment between the shoulders, hips, and ankles in a straight line. The free arm rests on the waist, and the free leg is positioned on the medial side of the supporting foot; (b) Level 2, raise the free leg and parallel to the floor; (c) Level 3, move forward the free leg as much as possible; (d) Level 4, move backward the free leg as much as possible; (e) Level 5 is the same as Level 1.

In the Five-Level Side Bridge test [Fig. 3 (a)-(e)], the participants initiated the test from a side-lying position, supporting themselves on one elbow and foot. The supporting elbow was flexed at 90°, and the leg was extended to maintain alignment between the shoulders, hips, and ankles in a straight line. The free arm rested on the waist, and the free leg was positioned on the medial side of the supporting foot. This position was maintained for 30 s [Fig. 3 (a)]. At Level 2, the free leg was raised parallel to the floor for 15 s [Fig. 3 (b)]. At Level 3, the free leg was moved forward as much as possible, holding for 15 s [Fig. 3 (c)]. At Level 4, the free leg was moved backward as much as possible, holding for 15 s [Fig. 3 (d)]. At Level 5, the free leg was returned to the starting position and maintained this position for 30 s [Fig. 3 (e)].

#### 2.3.2 Y Balance Tests

The Y Balance Test, encompassing the Lower Quarter and Upper Quarter Y Balance Tests, requires participants to use one side of their lower or upper limbs to move the indicators in three different directions as far as possible while maintaining their balance. This test challenges the participants’ proprioception, neuromuscular coordination, control, stability in movement, and strength [36,37] The further one reaches out, the higher the score will be received. Additionally, the test has been proven reliable, valid, and easy to perform [6,[38], [39], [40], [41]]. Therefore, it was used to evaluate dynamic core stability appropriately and comprehensively.

**Fig. 4:**
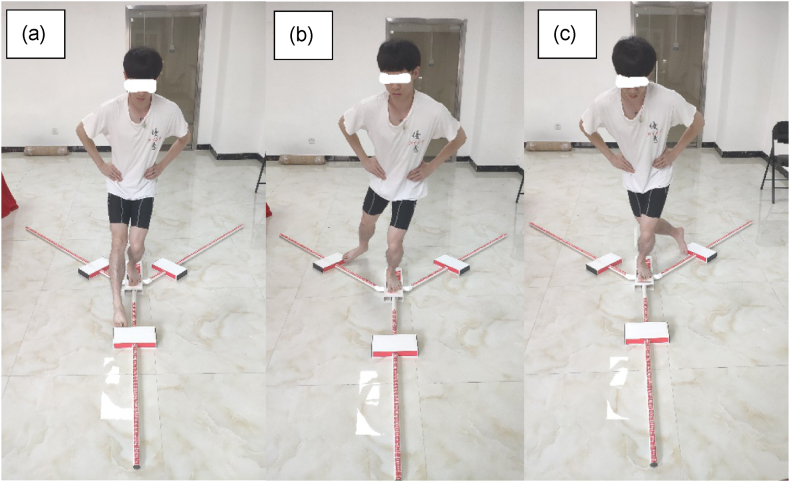
Lower Quarter Y Balance Test. (a) Anterior reach; (b) Posteromedial reach; (c) Posterolateral reach.

As no athletes were involved, the limb dominance effect should not have emerged in this study [42]. Therefore, only the dominant limbs were tested. For the Lower Quarter Y Balance Test [Fig. 4 (a)-(c)], the participants stood barefoot behind the red line of the stance platform. They started with six practice trials in each direction: anterior (A) [Fig. 4 (a)], posteromedial (PM) [Fig. 4 (b)], and posterolateral (PL) [Fig. 4 (c)]. After a 5-min break, an official test consisting of three testing trials for each direction was conducted. The participants were required to push the indicator as far as possible using their reaching foot and return to the starting position while maintaining balance with their hands on the hips. Using momentum to move the indicator, stepping on top of the indicator while pushing it, touching the ground, or losing balance (i.e. removing hands from the hips, failing to maintain a single stance, or failing to return to the initial position) were considered invalid trials. These data were discarded, and the trials were repeated.

**Fig. 5:**
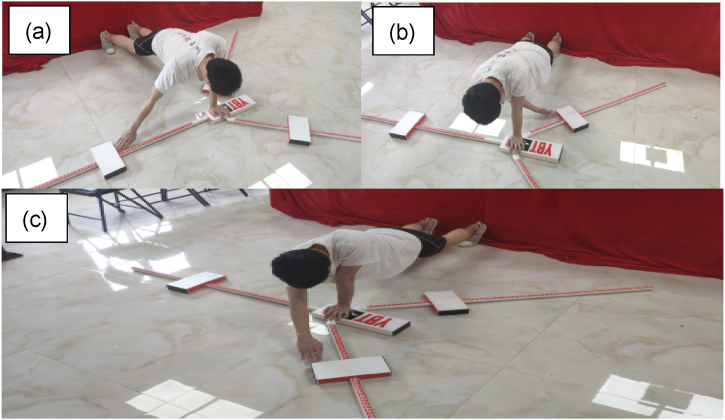
Upper Quarter Y Balance Test. (a) Medial reach; (b) Inferomedial reach; (c) Superomedial reach.

The Upper Quarter Y Balance Test [Fig. 5 (a)-(c)] was conducted after a 10-min break. The participants placed their testing hands on the stance platform, and their thumb was adducted while being aligned behind the red line. Similarly, the test began with six practice trials in each direction: medial (M) [Fig. 5 (a)], inferomedial (IM) [Fig. 5 (b)], and superomedial (SM) [Fig. 5 (c)]. After a 5-min break, an official test consisting of three trials for each direction was conducted. The participants were required to push the indicator as far as possible using their reaching hand while maintaining a push-up position with shoulder width apart. The trials were invalid and repeated if the participants failed to maintain balance (i.e. touched the ground or top of the indicator, failed to return to the initial position under control, lifted either foot off the ground), or failed to contact the target area of the indicator consistently while moving it.

Three research assistants supervised all the tests. Reaching distance was recorded to the nearest 0.5 cm. To avoid the influence of limb length differences, all raw data for reaching distance were normalised for analysis [36,43].

### 2.4 Bilateral and unilateral countermovement jump

Kinematic and kinetic data were simultaneously collected using an 8-camera Vicon Nexus system (VICON Corporation, Culver City, 4 CA, USA) operating at 200 Hz and an AMTI force plate (AMTI Corporation, Watertown, MA, USA) operating at 800 Hz. A three-dimensional model was constructed using Visual3D (C-Motion Inc., USA). A fourth-order zero-lag Butterworth filter with a cut-off frequency of 13 Hz was used to smooth and filter all kinematic data, whereas a cut-off frequency of 40 Hz was used for the kinetic data process. The jumping height was calculated using Equation (1):

(1) $$H={\left[\int \left(\frac{F\left(t\right)}{m}-g\right)dt\right]}^{2}/2g$$

All participants performed five trials each of the CMJ and SCMJ according to the instructional video guidance. During data collection, the participants executed three successful CMJ and three successful SCMJ trials (refer to studies by Avedesian et al. and others [25,28,[44], [45], [46]]). A trial was considered successful if the participant completed the jump and landed stably at the centre of the force plate. The average data from the three successful trials for each participant were used in the analysis. Otherwise, data from unsuccessful trials were excluded, and retests were conducted as necessary. A 2-min break was provided between each trial.

In this study, the angles and torques of the hip, knee, and ankle and their directions adhered to the general law of Visual3D: the x-axis represents flexion/extension, the y-axis represents adduction/abduction, and the z-axis represents internal/external rotation. Positive values indicated hip flexion/adduction/internal rotation, knee extension/adduction/internal rotation, and ankle dorsiflexion/varus/internal rotation.

#### 2.4.1 Division of vertical jump

**Fig. 6:**
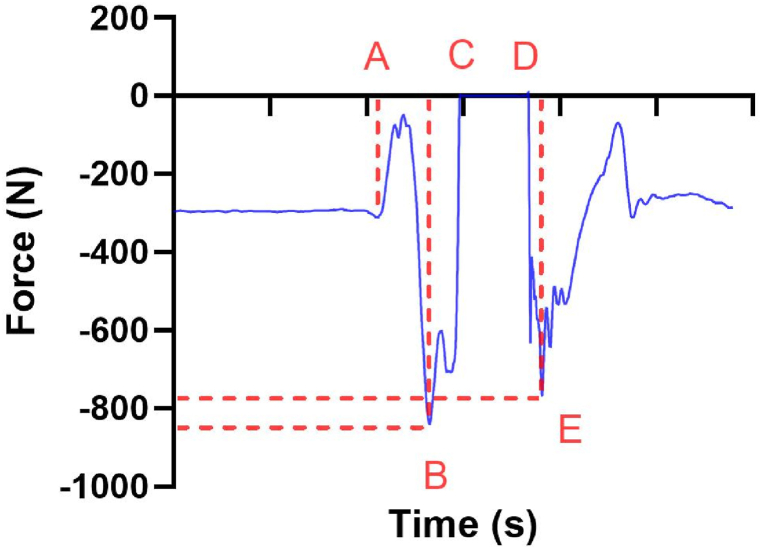
Plot for ground reaction force-time during the vertical jump.

The vertical jumps were divided into distinct phases. Significant changes in the force data marked the propulsive phase until it reached zero (Points A- C in Fig. 6, with Point B representing the maximum vertical ground reaction force during this phase). The flight phase was characterised by zero force data (Points C-D). The landing phase commenced when the force data significantly changed from zero, following the propulsive phase (after Point D, with Point E representing the maximum vertical ground reaction force during the landing phase).

### 2.5 Statistical analyses

Normality was assessed using the Shapiro–Wilk test. The relationship between the core stability and vertical jump measures was determined using the Pearson correlation coefficient (r). Interpretations of the correlation coefficient (r) were based on the following criteria [47]: no correlation or trivial correlation, |r|<0.2; fair, 0.2≤|r|＜0.4; moderate, 0.4≤|r|＜0.6; substantial, 0.6≤|r|＜0.8; and almost perfect, 0.8≤|r|. Statistical significance was set at p ≤ 0.05. All statistical tests were performed using SPSS (version 20.0., IBM, Chicago, USA).

## 3 Results

### 3.1 Correlations between core stability and the propulsive phase of CMJ

Table 1 displays the correlations between core stability and the biomechanical characteristics of CMJ during the propulsive phase. Main findings were summarized as follows.

**Table 1:** Correlations between core stability and biomechanical indexes of CMJ during the propulsive phase.

Parameters	YBT-LQ				YBT-UQ				Prone Bridge (s)	Side Bridge (s)
	A	PM	PL	Overall	M	IM	SM	Overall		
$\bar{F_{z}}$ （N）	−0.41	−0.47*	−0.44*	−0.52*	−0.02	−0.17	−0.16	−0.15	−0.14	−0.32
N $\bar{F_{z}}$ （N/kg）	−0.19	−0.05	−0.04	−0.12	0.26	0.01	−0.04	0.06	0.14	0.06
Δ $\bar{F_{x}}$ （N）	−0.19	−0.22	−0.26	−0.27	0.12	0.13	−0.09	0.06	−0.18	−0.52*
Δ $\bar{F_{y}}$ （N）	−0.09	−0.22	−0.17	−0.18	−0.09	−0.21	−0.12	−0.17	−0.55**	−0.49*
Δ $I_{x}$ （N·s）	−0.08	−0.17	−0.24	−0.20	0.04	0.11	−0.09	0.02	−0.25	−0.52*
Δ $I_{y}$ （N·s）	0.03	−0.14	−0.11	−0.08	−0.23	−0.20	−0.11	−0.20	−0.62**	−0.50*
NΔ $\bar{T_{ankle\;y}}$ (N·m/kg)	−0.21	0.06	−0.01	−0.08	−0.23	−0.46*	−0.29	−0.39	0.41	0.05
Δ $D_{left\;ankle\;y}$ (°)	−0.18	−0.47*	−0.44*	−0.43	−0.23	−0.50*	−0.43	−0.48*	0.33	0.12
Δ $D_{left\;ankle\;z}$ (°)	0.27	0.50*	0.49*	0.49*	0.32	0.52*	0.47*	0.52*	−0.25	−0.06
Δ $D_{right\;ankle\;y}$ (°)	0.09	−0.54*	−0.47*	−0.34	−0.51*	−0.44*	−0.35	−0.49*	−0.12	−0.35
Δ $D_{right\;ankle\;z}$ (°)	−0.15	0.47*	0.40	0.26	0.51*	0.45*	0.31	0.48*	0.13	0.32

1) *, p < 0.05; 2) **, p < 0.01; 3) $\bar{F}$: average force; 4) Δ $\bar{F}$ = $\left|\bar{F_{right\;}}-\bar{F_{left\;}}\right|$; 5) I: impulse; 6) Δ I = $\left|I_{right}-I_{left}\right|$; 7) $\bar{T}$: average torque; 8) $\Delta \bar{T}$ = $\left|\bar{T_{right}}-\bar{T_{left}}\right|$; 9) ΔD: changes of the joint angle; 10) N = normalised; 11) A: anterior reach; 12) PM: posteromedial reach; 13) PL: posterolateral reach; 14) M: medial reach; 15) IM: inferomedial reach; 16) SM: superomedial reach; 17) YBT-LQ: Lower Quarter Y Balance Test; 18) YBT-UQ: Upper Quarter Y Balance Test; 19) CMJ: countermovement jump.

Regarding static core stability, significantly negative correlations were observed between Eight-Level Prone Bridge Test and Δ $\bar{F_{y}}$ (r = −0.55 [moderate]), and Δ $I_{y}$ (r = −0.62 [substantial]). Similarly, significant negative correlations were observed between the Five-Level Side Bridge Test and Δ $\bar{F_{x}}$ (r = −0.52 [moderate]), Δ $\bar{F_{y}}$ (r = −0.49 [moderate]), Δ $I_{x}$ (r = −0.52 [moderate]), and Δ $I_{y}$ (r = −0.50 [moderate]).

Regarding dynamic core stability, significant correlations were observed between the PM reach of the Lower Quarter Y Balance Test and $\bar{F_{z}}$ (r = −0.47 [moderate]), Δ $D_{left\;ankle\;y}$ (r = −0.47 [moderate]), Δ $D_{left\;ankle\;z}$ (r = 0.50 [moderate]), Δ $D_{right\;ankle\;y}$ (r = −0.54 [moderate]), and Δ $D_{right\;ankle\;z}$ (r = 0.47 [moderate]). In addition, significant correlations were observed between the PL reach of the Lower Quarter Y Balance Test and $\bar{F_{z}}$ (r = −0.44 [moderate]), Δ $D_{left\;ankle\;y}$ (r = −0.44 [moderate]), Δ $D_{left\;ankle\;z}$ (r = 0.49 [moderate]), and Δ $D_{right\;ankle\;y}$ (r = −0.47 [moderate]). Significant correlations were observed between the overall score of the Lower Quarter Y Balance Test and $\bar{F_{z}}$ (r = −0.52 [moderate]), and Δ $D_{left\;ankle\;z}$ (r = 0.49 [moderate]). Additionally, significant correlations were observed between the M reach of the Upper Quarter Y Balance Test and Δ $D_{right\;ankle\;y}$ (r = −0.51 [moderate]) and Δ $D_{right\;ankle\;z}$ (r = 0.51 [moderate]). Significant correlations were observed between the IM reach of the Upper Quarter Y Balance Test and NΔ $\bar{T_{ankle\;y}}$ (r = −0.46 [moderate]) Δ $D_{left\;ankle\;y}$ (r = −0.50 [moderate]), Δ $D_{left\;ankle\;z}$ (r = 0.52 [moderate]), Δ $D_{right\;ankle\;y}$ (r = −0.44 [moderate]), and Δ $D_{righ\;ankle\;z}$ (r = 0.45 [moderate]). Furthermore, a significant positive correlation was observed between the SM reach of the Upper Quarter Y Balance Test and Δ $D_{left\;ankle\;z}$ (r = 0.47 [moderate]). Significant correlations were observed between the overall score of the Upper Quarter Y Balance Test and Δ $D_{left\;ankle\;y}$ (r = −0.48 [moderate])，Δ $D_{left\;ankle\;z}$ (r = 0.52 [moderate])，Δ $D_{righ\;ankle\;y}$ (r = −0.49 [moderate]), and Δ $D_{righ\;ankle\;z}$ (r = 0.48 [moderate]).

There was no significant correlation between core stability and the height performance of CMJ.

### 3.2 Correlations between core stability and the propulsive phase of SCMJ

Table 2 displays the correlations between core stability and the biomechanical characteristics of SCMJ during the propulsive phase. Main findings were summarized as follows.

**Table 2:** Correlations between core stability and biomechanical indexes of SCMJ during the propulsive phase.

Parameters	YBT-LQ				YBT-UQ				Prone Bridge (s)	Side Bridge (s)
	A	PM	PL	Overall	M	IM	SM	Overall		
$\bar{F_{left\;z}}$ （N）	−0.11	−0.47*	−0.51*	−0.43	−0.44	−0.20	−0.12	−0.27	−0.30	−0.30
N $\bar{F_{left\;z}}$ （N/kg）	0.19	−0.05	−0.15	0.00	−0.12	−0.04	0.06	−0.03	−0.20	0.04
N $\bar{T_{left\;ankle\;z\;}}$ (N·m/kg)	0.33	0.46*	0.54*	0.54*	0.28	0.22	0.18	0.25	−0.07	−0.15
$\Delta D_{left\;ankle\;y}$ (°)	0.20	−0.15	−0.04	0.03	0.09	0.14	0.12	0.14	0.52*	0.59**
$\Delta D_{left\;knee\;z}$ (°)	0.32	−0.05	0.01	0.13	0.27	0.31	0.31	0.34	0.49*	0.57**

1) *, p < 0.05; 2) **, p < 0.01; 3) $\bar{F}$: average force; 4) Δ $\bar{F}$ = $\left|\bar{F_{right\;}}-\bar{F_{left\;}}\right|$; 5) I: impulse; 6) Δ I = $\left|I_{right}-I_{left}\right|$; 7) $\bar{T}$: average torque; 8) $\Delta \bar{T}$ = $\left|\bar{T_{right}}-\bar{T_{left}}\right|$; 9) ΔD: changes of the joint angle; 10) N = normalised; 11) A: anterior reach; 12) PM: posteromedial reach; 13) PL: posterolateral reach; 14) M: medial reach; 15) IM: inferomedial reach; 16) SM: superomedial reach; 17) YBT-LQ: Lower Quarter Y Balance Test; 18) YBT-UQ: Upper Quarter Y Balance Test; 19) SCMJ: single countermovement jump.

Regarding static core stability, significantly positive correlations were observed between the Eight-Level Prone Bridge Test and $\Delta D_{left\;knee\;z}$ (r = 0.49 [moderate)]). Similarly, significantly positive correlations were observed between the Five-Level Side Bridge Test and Δ $D_{left\;ankle\;y}$ (r = 0.59 [moderate]) and $\Delta D_{left\;knee\;z}$ (r = 0.57 [moderate]).

Regarding dynamic core stability, significant correlations were observed between the PM reach of the Lower Quarter Y Balance Test and $\bar{F_{left\;z}}$ (r = −0.47 [moderate]) and N $\bar{T_{left\;ankle\;z\;}}$ (r = 0.46 [moderate]). Similarly, significant correlations were observed between the PL reach of the Lower Quarter Y Balance Test and $\bar{F_{left\;z}}$ (r = −0.51 [moderate]) and N $\bar{T_{left\;ankle\;z\;}}$ (r = 0.54 [moderate]). Additionally, a significant positive correlation was observed between the overall Lower Quarter Y Balance Test score and N $\bar{T_{left\;ankle\;z\;}}$ (r = 0.54 [moderate]).

There was no significant correlation between core stability and the height performance of SCMJ.

## 4 Discussion

This study aimed to re-examine the correlation between core stability and vertical jump performance. To the best of our knowledge, this is the first study to measure core stability using the Eight-Level Prone Bridge, Five-Level Side Bridge, and Y Balance Tests to decipher the relationship between core stability and vertical jump from the perspective of biomechanics. Based on the results of this study, the correlation analysis did not reveal any significant correlations between core stability and the height performance of CMJ or SCMJ. These findings support the results of previous studies by Parkhouse et al. and others [5,10,12,19].

For instance, by applying plank and double leg lowering as static core tests and back extension as the dynamic core test, Parkhouse et al. [12] reported that an increased core stability did not result in improved outcome performance in vertical jumps, sprints, or medicine ball throws. This lack of correlation can be revealed once the analysis delves into the determinants of jumping height. According to Equation (1), the jumping height (H) is determined by the normalised ground reaction force and time. Given that no correlations were observed between the normalised ground reaction force and core stability, a significant correlation between core stability and H would only emerge if the time of force application was prolonged. However, vertical jumping is classified as an explosive sport, emphasising its ability to generate significant force or energy quickly. In contrast, core stability primarily focuses on maintaining stability during static or dynamic movements. Prolonging the action time can potentially hinder the effective generation of force and, in turn, impair the ability to produce an explosive force. This notion is supported by the negative correlations observed between the core stability and ground reaction force.

According to our results, PM reach, PL reach, and the overall score of the Lower Quarter Y Balance Test had moderately negative correlations with $\bar{F_{z}}$ (r = −0.47, −0.44, and −0.52 respectively) in CMJ. Similarly, the PM reach and PL reach of the Lower Quarter Y Balance Test had moderately negative correlations with $\bar{F_{left\;z}}$ (r = −0.47 and −0.51 respectively). This indicates that a stronger core stability does not benefit but may have a harmful effect on explosive force generation. This finding explains why some studies that have observed improvements in core stability cannot translate them into outcome performance improvements [12,48]. The mutual restriction between force and time is likely to be the key factor limiting the translation of improved core stability into height performance. In this regard, it is reasonable to assume that increased core stability may be insufficient to enhance the outcome performance of vertical jumps and other explosive sports events [16,23] due to impaired explosive force generation.

However, it can be concluded that the contribution of core stability to vertical jump performance based merely on the evidence mentioned above from the outcome performance (jumping height) is not entirely accurate. This is because vertical jump performance comprises not only outcome performance but also process performance. Moreover, the outcome performance is determined by the process performance. This has been neglected in previous studies [12,16,23]. Therefore, they tend to conclude the correlation between core stability and vertical jump performance or pose practical implications given the correlation between core stability and outcome performance (e.g., no correlation). For example, Okada et al. [23] suggested that core stability should not be the primary emphasis of any training programme, given the undesirable correlation between core stability and outcome performance. However, different attitudes or findings should be considered when delving into the process analysis of vertical jumps. Although core stability cannot trigger improved height performance, other biomechanical factors displaying significant correlations with core stability indicate that core stability refines vertical jump performance. In other words, core stability should benefit the process performance of vertical jumps, namely, movement quality-- the manner of optimisation when performing a specific movement pattern. This is a prerequisite for future improvements in outcome performance and injury prevention [49].

Specifically, during the propulsive phase of CMJ, moderately to substantially negative correlations were observed between static core stability and kinetic indicators related to differences in the force applied to both sides of the limbs (e.g. Δ $\bar{F_{x}}$, Δ $\bar{F_{y}}$, Δ $I_{x}$, and Δ $I_{y}$). These findings suggest that individuals with greater static core stability can achieve the goal of a more balanced and coordinated power generation. Similarly, moderately negative correlations between dynamic core stability and NΔ $\bar{T_{ankle\;y}}$, $\Delta D_{left\;ankle\;y}$ and Δ $D_{right\;ankle\;y}$ in CMJ, moderately positive correlations between dynamic core stability and Δ $D_{left\;ankle\;z}$ and Δ $D_{right\;ankle\;z}$ in CMJ, moderately positive correlations between dynamic core stability and N $\bar{T_{left\;ankle\;z\;}}$ in SCMJ, and moderately positive correlations between static core stability and $\Delta D_{left\;knee\;z}$ in SCMJ suggest that greater core stability helps maintain a reasonable posture during movement or an optimal jumping pattern. In other words, individuals with greater core stability tend to experience ankle valgus and internal rotation during jumping. These are similar to the patterns observed in the Y Balance Test, where participants must centre their body weight within the support base for balance while performing movements in three different directions.

Hence, the correlations mentioned above indicate that individuals with better core stability should experience improved process performance of vertical jumps, as these individuals experience a more balanced force load and a more optimal and reasonable movement posture. Furthermore, this optimal movement quality brought about by core stability provides a foundation for possible jumping height improvements.

Overall, increased core stability should improve vertical jump performance. However, this improvement is insufficient to translate into outcome performance enhancement given the limited contribution of core stability to explosive force generation.

This study had some limitations. First, the limited sample size may be one of the potential reasons for the impaired correlation between core stability and vertical jump. Future studies should consider recruiting larger sample sizes or determining sample sizes based on statistical calculations. Second, the exercise frequency and duration were self-reported. Third, the findings of this study may not be generalisable to a broader population, including women and individuals of different age groups. For example, women may exhibit different biomechanics, neuromuscular control, and risk factors for injuries. Therefore, the applicability of current core stability tests for women remains uncertain. Further studies using diverse samples are required to obtain a more comprehensive understanding. Finally, this study solely focused on vertical jumps as a representative of explosive sports events. Additional studies involving sports with different characteristics, such as endurance events or other types of explosive sports, are required to gain more comprehensive insight into the effects of core stability on sports performance.

Coaches should leverage the benefits of incorporating core stability training into specialised training when refining vertical jump techniques is the principal problem. This would be helpful in providing sports enthusiasts with the opportunity to observe future improvements in athletic performance. However, core stability training should not be applied to individuals with relatively outstanding movement quality or excellent vertical jump athletic performance because it may impair their explosive characteristics.

## 5 Conclusion

This study delved deeper into understanding the correlation between core stability and vertical jumps, going beyond the narrow focus of jumping height alone. Moderate to substantial correlations between core stability and force difference and joint degree or torque changes indicate the beneficial effects of core stability on vertical jump performance given an improved movement quality. However, this benefit brought about by core stability may be insufficient to improve height performance, owing to impaired explosive force generation.

## Research ethics approval

This study was approved by the Human Research Ethics Committee of South China Normal University (SCNU-SPT-2020-010).

## Consent to participate

Participants received comprehensive instructions regarding the study procedures and expectations, and they were given written informed consent before the experiment. All methods were carried out in accordance with the Helsinki legislation.

## Data availability statement

Data included in article/supp. Material/referenced in the article. Data will be made available on reasonable request.

## Disclosure of interest

The authors declare that they have no known competing financial interests or personal relationships that could have appeared to influence the work reported in this paper.

## CRediT authorship contribution statement

**Jay Lee:** Writing – review & editing, Writing – original draft, Methodology, Formal analysis, Conceptualization. **Liang Wang:** Writing – review & editing, Validation, Supervision, Conceptualization. **Xiuli Zhang:** Writing – review & editing, Validation, Supervision, Conceptualization.

## Declaration of competing interest

The authors declare that they have no known competing financial interests or personal relationships that could have appeared to influence the work reported in this paper.
